# Comparison of leucine-rich alpha-2-glycoprotein-1 (LRG-1) plasma levels between patients with and without appendicitis, a case–controlled study

**DOI:** 10.1038/s41598-021-84013-2

**Published:** 2021-03-10

**Authors:** Marcelo Bentancor Lontra, Ricardo F. Savaris, Leandro Totti Cavazzola, Jackson Maissiat

**Affiliations:** 1 Postgraduate Program in Medicine: Surgical Sciences, School of Medicine, Universidade Federal do Rio Grande do Sul, Porto Alegre, 90035-002 Brazil; 2 Surgical Oncologist and General Surgeon, Military Hospital of Porto Alegre, Porto Alegre, RS 90440-191 Brazil; 3 Surgical Oncologist and General Surgeon, Moinhos de Vento Hospital, Porto Alegre, RS 90035-000 Brazil; 4 Department of Obstetrics and Gynecology, School of Medicine, Universidade Federal do Rio Grande do Sul, Porto Alegre, RS 90035-002 Brazil; 5 Associate Professor of Surgery, Department of Surgery, School of Medicine, Universidade Federal do Rio Grande do Sul, Porto Alegre, RS 90035-002 Brazil

**Keywords:** Diagnosis, Biomarkers

## Abstract

Acute appendicitis (AA) is the first cause of emergency surgery. Leucine-Rich Alpha-2-Glycoprotein 1 (LRG1) has been shown to be a potential biomarker in cases of AA in children, but there are conflicting results for its use in adults. The objective of this study is to compare the median plasma values of LRG1 in patients with acute abdomen with and without appendicitis. This case–control study was conducted prospectively at the emergency room (ER) of a tertiary teaching hospital, between March 1st, 2011 and December 31st, 2012. Patients with recent abdominal pain, aged 18–70 years who attended at the ER were included in the study. Blood samples were drawn at the first presentation. Those who were submitted to surgery and had a pathology report of AA were considered as cases. Those without a need for surgery and treated for other conditions, e.g., pelvic inflammatory disease, were considered as controls. Follow-up in controls was made up to 30 days. LRG1 plasma median values were measured using an ELISA kit and compared between groups. A total of 28 participants, 14 cases with acute appendicitis and 14 controls, were included. The median (range) values of leucine-rich alpha-2-glycoprotein-1 level in the group with appendicitis and control group were 8.8 ng/ml (5.5–31) and 11 (4.6–108) ng/ml, respectively (Mann–Whitney test P = 0.26). Median plasma leucine-rich alpha-2-glycoprotein-1 levels were not useful in diagnosing Acute Appendicitis in patients with acute abdominal pain.

## Introduction

Acute appendicitis (AA) is the first cause of emergency surgery in the United States, with about 250,000 cases per year^1^ and has a peak incidence in the 10–24-years-old age group; the incidence in men is higher than in women^2^. Obstruction of the lumen of the appendix (fecalith, normal stool, or lymphoid hyperplasia) is the main cause of acute appendicitis. Retrospective appendectomy data suggest fecalith prevalence of 18% among patients with an emergency^3,4^. Appendicitis continues to be a condition at high risk for missed and delayed diagnosis. Negative appendectomy rates range between 5 and 40%, while late operative intervention occurs between 5 and 30%^5^. There is no single historical or physical finding or laboratory test that can definitively make the diagnosis^6^. Patients with appendicitis continue to display a complex and atypical range of clinical manifestations, providing a subsequent high risk for emergency physicians to miss acute abdominal pathology on a patient's initial visits^7^.

Many authors have been searching for new biomarkers for diagnosing appendicitis^8^. Among these new biomarkers, there is the leucine-rich alpha-2-glycoprotein 1 (LRG1). LRG1 is part of a leucine-rich repeating-proteins family that has involvement with double-protein interactions, cell adhesion, signal transduction, and acute inflammation of bacterial infection^9,10^. The use of LRG1 for diagnosing appendicitis was first reported in 2010^11^. These authors used mass spectrometry with a High-Pressure Liquid Chromatography system in the exploratory phase and western blotting in the validation phase. Validation was performed using urine samples from 67 children with abdominal pain; from these, 25 children had a confirmed case of appendicitis. A ROC curve analysis revealed that LRG1 had an area under the curve of 0.97. Later, a proper prospective method found that urinary and serum levels of LRG1 were higher in children with a diagnosis of appendicitis^12^. In 2012, other authors reported limitations on urinary ELISA assays, despite the discrimination between patients with and without acute appendicitis; these authors suggested that a clinical-grade urinary LRG1 assay was necessary^13^. In 2016, a research group reported that urinary LRG1, when adjusted to dehydration, would be promising novel biomarkers for appendicitis in children^14^. Finally, in 2017, using an adult population (> 18 years-old) and a commercial ELISA kit for LRG1, Rainer et al*.* found a significant difference in plasma median levels of LRG1 between patients with and without appendicitis. However, after using logistic regression analysis, plasma levels of LRG1 were not significant to discriminate acute appendicitis^15^. Likewise, Dermici et al. reported that plasma levels of LRG1 were not useful to differentiate between acute appendicitis and pelvic inflammatory disease in women with acute abdominal pain in right lower-quadrant^16^.

Thus, due to the great variability of kits and to the scant data on the accuracy of LRG1 in an adult population with acute abdominal pain, it is necessary to investigate with different ELISA kits to evaluate the accuracy of LRG1 in the differential diagnosis of acute appendicitis.

The objective of this study is to compare the plasma levels of LRG1 in an adult population with and without the diagnosis of appendicitis, using a commercial ELISA kit.

## Results

### Participants

Between March 1st, 2011 and December 31st, 2012, 28 cases who presented at the emergency room at HCPA with a hypothesis of appendicitis and with inclusion criteria were enrolled.

### Descriptive data

The characteristics of the studied population are in Table 1. Briefly, there were no significant differences between groups when age, sex, ethnicity and the presence of fever were compared.

**Table 1:** Characteristics of the studied population.

Characteristics	Appendicitis n = 14	No appendicitis n = 14	p value
Age (years-old)—mean (SD)	40.7 (16.1)	32.7 (13.1)	0.09^a^
**Sex—n (%)**			
Male	6 (43.9)	3 (21.4)	0.41^b^
Female	8 (57.1)	11 (78.6)	
**Ethnicity—n (%)**			
Caucasian	12 (85.7)	10 (71.4)	0.65^b^
Non-caucasian	2 (14.3)	4 (28.6)	
Days from initial symptoms—median (range)	2 (1–5)	4.5 (1–10)	0.04^a^
**Nausea—n (%)**			
Yes	9 (64.3)	7 (50.0)	0.70^b^
No	5 (35.7)	7 (50.0)	
Fever—n (%)			
Yes	4 (28.6)	1 (7.1)	0.33^b^
No	10 (71.4)	13 (92.9)	
**Pain migrating—n (%)**			
Yes	7 (50.0)	0 (0.0)	0.006^b^
No	7 (50)	14 (100.0)	
**Rebound tenderness—n (%)**			
Yes	5 (35.7)	1 (7.1)	0.17^b^
No	9 (64.3)	13 (92.9)	
Leukocytes count, 1000 cells/mm^3^—mean (SD)	13.2 (5.9)	7.8 (2.4)	0.006^a^
**Final diagnosis**			
Appendicitis	14 (100)		
Normal appendix (visual)		2	
Normal appendix (pathology)		1	
Spontaneous resolution		8	
Hemorrhagic ovary cyst		1	n.a
Pelvic inflammatory disease		1	
Mesenteric lymphadenitis		1	

*n.a.* not applicable.

^a^Mann–Whitney.

^b^Fisher Exact test.

Patients with appendicitis had a higher incidence of pain migration to the lower right quadrant and higher mean leukocyte count. Further details are in the “Supplement material S1”.

### Main outcomes

A total of 14 cases of appendicitis were confirmed by a pathology report. Non-appendicitis cases comprised 14 patients. The main diagnosis was spontaneous resolution.

**Figure 1:**
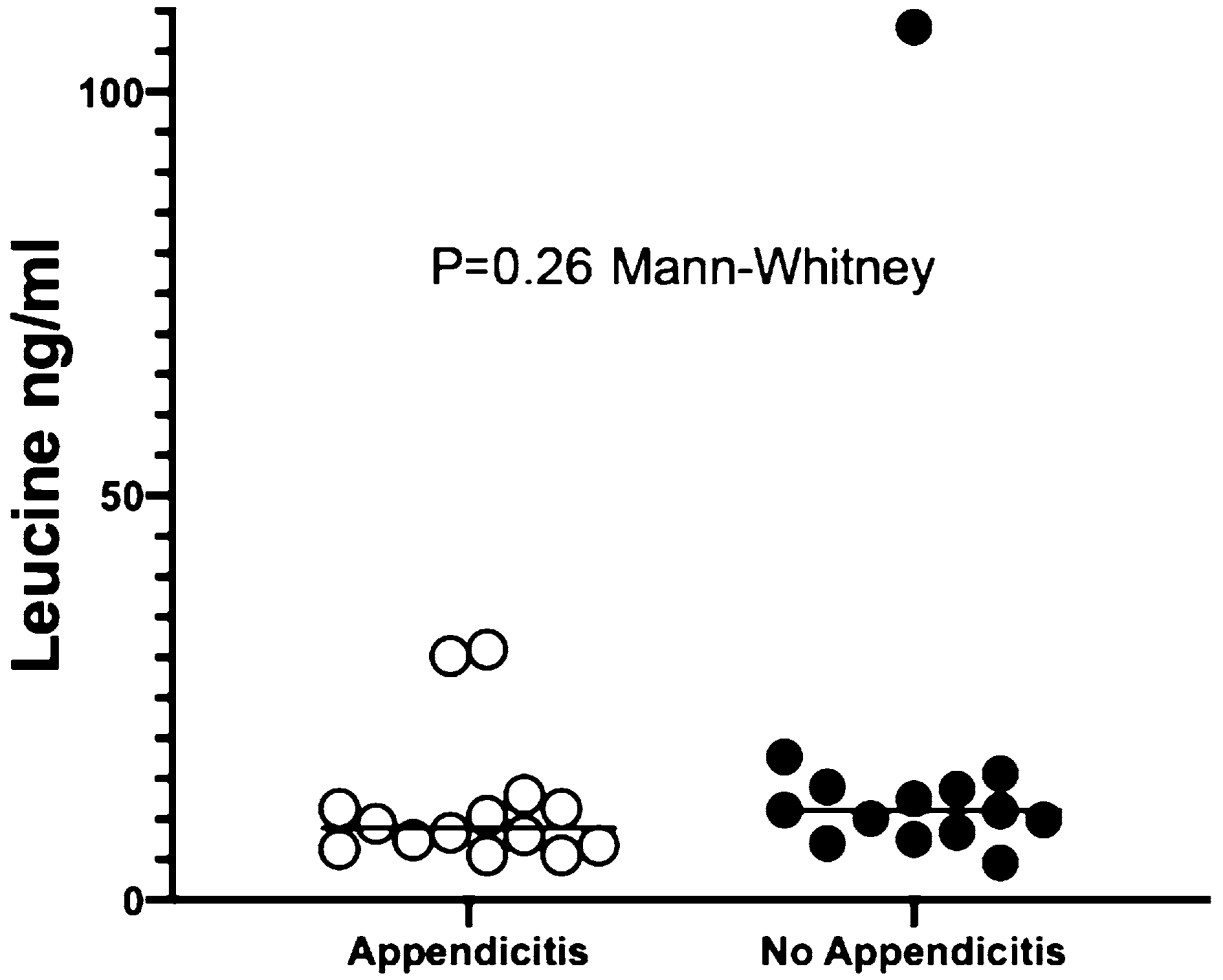
Plasma levels of leucine (LRG1) in patients with and without appendicitis.

Median (range) plasma levels of LRG1 were 8.8 ng/ml (5.5–31) and 11 (4.6–108) ng/ml, in cases with and without appendicitis, respectively (P = 0.26, Mann–Whitney U-test), as shown in Fig. 1.

## Discussion

No difference was found between plasma levels of patients with and without appendicitis, using the present commercial kit. Our results are different from those reported by Rainer et al.^15^. Although we also found that median levels were lower in patients with acute appendicitis, we were not able to find a significant difference between these values (Fig. 1), mainly due to the wide range of the results presented in cases without appendicitis (range from 4.6 to 108 ng/ml). One possibility could be related to the difference in the ELISA kit used or to our non-appendicitis group. In our study, the majority of controls had a spontaneous resolution, some had a pelvic inflammatory disease or mesenteric lymphadenitis, which are acute inflammation and may elevate LRG1^9,10^. We are not able to make a further comparison to the original work of Rainer's study since there are no details of the final diagnosis of the non-appendicitis cases^15^.

The limitations of this study are: (1) we used only one commercial ELISA kit, (2) samples were kept in − 80 °C for 3 months (we are not aware that this time may alter the protein expression), and (3) although we performed a proper sample size calculation, a post hoc analysis identified that the standard deviation was properly identified, but the difference between both groups was minimal, i.e., 1.2 ng/ml; so, such small differences would require a larger sample; clinical significance, however, needs further analysis.

The use of confirmed cases of appendicitis using the pathology report as the gold standard and the proper follow-up with those who had a hospital discharge without surgery are strengths of the study. In addition, we presented a representative population, with some differential diagnosis in the control group.

The current ELISA method used herein was not applicable to distinguish cases with and without appendicitis. Analysis with mass spectrometry is still preferable^11,13,17^, but it does not seem feasible. There is a need to confirm the results by other centers and using different ELISA kits before implementing LRG1 as a biomarker for diagnosing appendicitis.

In conclusion, our findings show that median plasma levels of leucine-rich alpha-2-glycoprotein 1 are not different between cases with and without acute appendicitis.

## Methods

### Study design

This is a case–control study conducted at the emergency room in a tertiary teaching hospital, between March 1st, 2011 and December 31st, 2012, following the STROCSS criteria^18^.

### Participants

All patients presented in the adult emergency unit of HCPA with acute abdominal pain, with a diagnostic hypothesis of appendicitis, were potentially eligible for this study.

The inclusion criteria were recent abdominal pain (< 30 days) with at least one more of the following historical/clinical findings: pain in the right iliac region, pain migrating, pain before vomiting, psoas sign, fever, rebound tenderness at Mcburney's point (Blumberg sign), abdominal rigidity, no previous similar pain, pain to digital rectal examination associated or not to anorexia, nausea or vomiting.

Patients that declined to participate in the study or who already had an appendectomy were excluded.

### Variables

Plasma levels of LRG1 (ng/ml) were our main index test. Pathology report of the appendix specimen, in those who went to surgery and had their appendix removed, was the reference standard for diagnosis.

Controls, i.e., patients without appendicitis, were defined by those who had a normal appendix, either by pathology or surgery, or a different outcome on a 30 days follow-up, e.g., spontaneous resolution of the abdominal pain or pelvic inflammatory disease. The 30 days follow-up was performed by contacting the patient by telephone.

### Data sources/measurements

Data were prospectively obtained from patients who attended our emergency unit with a suspected diagnosis of appendicitis, according to our study design.

Blood samples were collected using EDTA vacuum tubes in the first consultation. Samples were centrifuged for 15 min at 1000 × *g* at 4 °C within 30 min of collection. Plasma supernatant was harvested and stored at − 80 °C for posterior ELISA analysis.

### LRG1 ELISA quantification

Samples were thawed and submitted to ELISA quantitative analysis using Human-LRG1 ELISA-Kit (MyBiosource; Catalog No: MBS2502648, Inc., San Diego, CA, USA), according to the instructions of the manufacturer. The kit had a minimum detectable dose of LRG1 of 4.688 ng/ml, although the usual detection range varies between 7.813 and 500 ng/ml. The coefficient of variation of repeatability was less than 10%. This kit recognizes natural and recombinant Human LRG1. No significant cross-reactivity or interference between human LRG1 and analogs was observed.

ELISA microplates were read using SpectraMax M3 (Molecular Devices, LLC, San Jose, CA, USA).

### Bias

In order to reduce bias, all ELISA measurements were performed in duplicates in one batch.

### Sample size

The sample size was calculated according to the literature^19^. At least 28 patients (14 cases in each group) were required to have a 95% chance of detecting, as significant at the 1% level, an increase in the median plasma level of LRG1 measure from 53 ng/ml in the non-appendicitis group to 95 ng/ml in the experimental group (appendicitis group), based on the data previously published at literature^12^. The standard deviation of 26 was obtained from an in-house pilot study.

### Quantitative variables

Plasma levels of LRG1 were measured in ng/ml, age were measured in years and leukocyte count by number of cells/mm^3^.

### Statistical methods

The normal distribution of LRG1 plasma levels was analyzed using the D'Agostino & Pearson test. Samples that passed the normality test were analyzed with Student t-test, otherwise, the Mann–Whitney U-test was applied. Dichotomic data were analyzed using Fisher's exact test. Statistical analysis was performed using GraphPad Prism software version 8.0 (GraphPad Software, La Jolla, CA, USA).

### Statements and ethical aspects

Marcelo Bentancor Lontra, Jackson Maissiat, Leandro Totti Cavazzola and Ricardo Francalacci Savaris declare that they have no conflicts of interest. All methods and experiments applied here were carried out in accordance with relevant guidelines and regulations. All participants consented to use their results of laboratory tests for research, once written informed consent was obtained from all subjects of this study. All experimental protocols, as well as the ethics procedures to use any human data, were approved by the local research ethics committee of HCPA under CAAE 01119.0.001.000-11 and registered at ReBEC (http://www.ensaiosclinicos.gov.br/) under the number *RBR-9dxqbk*. Patients were not involved in the study design.

## Supplementary Information

Supplementary Information.
