# Supplementary material for: Comparison of leucine-rich alpha-2-glycoprotein-1 (LRG-1) plasma levels between patients with and without appendicitis, a case–controlled study
Source: Sci Rep. 2021 Mar 10;11:5574. doi: 10.1038/s41598-021-84013-2 (PMC7946883; doi:10.1038/s41598-021-84013-2)
Supplement: Supplementary file 1 — Supplementary Information. [file 41598_2021_84013_MOESM1_ESM.docx]

**Supplemental Material** is available at this link:

<https://docs.google.com/spreadsheets/d/1Y9fJtU_YXqMyUrS5q3OHT9K0EdAIaHtlH58KZkYIjLw/edit?usp=sharing>
